# Molecular Profile (Estrogen Receptor, Progesterone Receptor, Bcl-2 and Ki-67) of the Ectopic Endometrium in Patients with Endometriosis

**DOI:** 10.3390/ijms26072983

**Published:** 2025-03-25

**Authors:** Ciprian-Andrei Coroleucă, Cătălin-Bogdan Coroleucă, Ruxandra Coroleucă, Petre Cornel Brătilă, Aniela-Roxana Nodiți, Ioana Roșca, Lăcrămioara Aurelia Brîndușe, Elvira Brătilă, Mihaela Boț

**Affiliations:** 1 Obstetrics and Gynecology Department, Faculty of Medicine, “Carol Davila” University of Medicine and Pharmacy, 050474 Bucharest, Romania; ciprian.coroleuca@umfcd.ro (C.-A.C.); pbratila49@yahoo.com (P.C.B.); elvira.bratila@umfcd.ro (E.B.); mihaela.bot@umfcd.ro (M.B.); 2 “Prof. Dr. Panait Sîrbu” Obstetrics and Gynecology Hospital, 060251 Bucharest, Romania; ccoroleuca@yahoo.com; 3 Ophthalmology Department, Faculty of Medicine, “Carol Davila” University of Medicine and Pharmacy, 050474 Bucharest, Romania; ruxandra.coroleuca@umfcd.ro; 4 “Euroclinic–Regina Maria” Hospital, 014452 Bucharest, Romania; 5 Surgery Department, Faculty of Medicine, “Carol Davila” University of Medicine and Pharmacy, 050474 Bucharest, Romania; aniela.noditi@umfcd.ro; 6 “Prof. Dr. Alexandru Trestioreanu” Institute of Oncology–Surgical Oncology, 022328 Bucharest, Romania; 7 Faculty of Midwifery and Nursery, “Carol Davila” University of Medicine and Pharmacy, 050474 Bucharest, Romania; 8 Department of Public Health and Management, Faculty of Medicine, “Carol Davila” University of Medicine and Pharmacy, 050474 Bucharest, Romania; lbrinduse@gmail.com; 9 “Elias” University Emergency Hospital, 011461 Bucharest, Romania

**Keywords:** endometriosis, biomarker, immunohistochemistry, laparoscopic treatment, estrogen receptor, progesterone receptor, Bcl-2, Ki-67

## Abstract

Endometriosis is characterized by alterations of the action and control mechanisms that lead to the development of ectopic endometrial tissue. This study aimed to analyze the molecular profile of ectopic endometrium by evaluating the expression of several biomarkers [estrogen receptor (ER), progesterone receptor (PR), anti-apoptotic protein Bcl-2, and Ki-67 antigen] in relation to the stage of the disease and symptoms. A prospective study over a period of one year, consisting of 14 patients with endometriosis, was performed. All patients received laparoscopic surgical treatment for excision of the lesions and staging of the disease. The expression of the aforementioned biomarkers was assessed in the ectopic endometrial tissue from the excised lesions using immunohistochemistry to determine their expression in the glandular epithelium and stroma. The mean expression of biomarkers in the epithelial and stromal levels did not differ significantly based on disease stage. Epithelial ER expression was significantly positively correlated with stromal ER, epithelial PR, and stromal PR. Stromal ER was significantly positively correlated with epithelial PR and stromal Ki-67. Epithelial Bcl-2 was significantly positively correlated with stromal Bcl-2. Epithelial Ki-67 was significantly positively correlated with stromal Ki-67. Finally, epithelial Bcl-2 expression was significantly positively correlated with the intensity of dyspareunia. The correlation between epithelial Bcl-2 expression and the intensity of dyspareunia highlights a potential molecular link to the severity of symptoms in endometriosis. These results suggest that further exploration of these biomarkers could lead to improved understanding of their clinical implications and more personalized therapies for patients with endometriosis.

## 1. Introduction

Endometriosis is a benign chronic inflammatory condition characterized by the appearance and development of endometrial tissue (endometrial glands and endometrial stroma) outside the uterus [1]. Endometriosis primarily affects women of reproductive age, among whom it has an incidence of 2–10% [2]. With a lower incidence, endometriosis is also found in adolescent girls and menopausal and postmenopausal women [3]. The etiology and pathophysiology of endometriosis are incompletely understood, despite extensive research efforts [4].

The symptoms of endometriosis are varied and frequently non-specific [5]. Although it can be asymptomatic, the clinical picture of endometriosis is frequently dominated by one or more types of pelvic pain (dysmenorrhea, dyspareunia, chronic pelvic pain, dysuria, or dyschezia) and infertility [5,6].

Considering the variability of the symptoms and the multitude of lesions, there is a need to adopt an endometriosis classification system recognized and used internationally [7]. The lack of a general consensus regarding classification has resulted in the establishment of several endometriosis classification systems. The most popular and widely used classification system for endometriosis is the one proposed by the American Society for Reproductive Medicine, introduced in 1979, and revised in 1985 and 1996 when it was renamed as the revised American Society for Reproductive Medicine (rASRM) classification system [7]. The rASRM classification system is based on intraoperative findings and quantifies peritoneal endometriotic lesions, ovarian endometriosis, Douglas posterior cul-de-sac obliteration, and ovarian and tubal adhesions [7]. Since the rASRM classification system does not consider retroperitoneal endometriotic lesions, the ENZIAN classification of deep infiltrative endometriosis was introduced in 2005 and was successively revised in 2010, 2011, and 2021 [7,8]. The ENZIAN classification includes the assessment of deep endometriotic lesions that affect the vagina, rectovaginal septum, uterosacral ligaments, parameters, rectum, and potentially other organs (uterus, bladder, ureter, other intestinal segments, lungs, diaphragm, and inguinal region) [7,8]. These classification systems are not competitors but are considered complementary, to describe as accurately as possible, the location and extent of endometriosis [9].

Endometriosis is an estrogen-dependent disease as estrogen influences the pathophysiological mechanisms of this condition [10]. The ectopic endometrium (endometrial tissue outside the uterine cavity) in endometriotic lesions reacts differently to estrogen and progesterone compared to eutopic endometrium (endometrial tissue) [11]. Alterations in the hormonal profile of the ectopic endometrial tissue can lead to increased potential for adhesion, proliferation, and protection against the host’s immune system in endometriotic lesions [12,13]. Endometriosis is associated with the alteration of the regulation and mechanistic actions of both estrogen and progesterone, which leads to the development of a hyperestrogenic state in conjunction with a state of progesterone resistance [14]. Estrogen receptors (ER) and progesterone receptors (PR) are the primary steroid receptors involved in the pathological mechanisms of endometriosis and at the same time represent the primary therapeutic targets of the hormonal treatment in this pathology [10,15]. Determination of the ER and PR expression profile in the lesions allows a personalized treatment for each patient with endometriosis [10,15].

Endometriosis causes the aberrant development of ectopic endometrial tissue by affecting the mechanisms that regulate apoptosis and cell proliferation [16,17]. Apoptotic mechanisms are altered in endometriosis [16]. Ectopic endometrium from superficial peritoneal lesions and endometriotic ovarian cysts are characterized by reduced levels of apoptosis, and thus, increased levels of anti-apoptotic B-cell lymphoma-2 (Bcl-2) protein expression levels [16]. Considering the proliferative nature of endometriosis, this pathology can be analyzed from the perspective of Ki-67 antigen expression, which is associated with cell proliferation [17]. Previously published studies highlighted the correlation between Ki-67 antigen levels and the stage of endometriosis [18,19]. The detection of Bcl-2 protein and Ki-67 antigen expression allows for an assessment of the aggressiveness and the recurrence capacity on a more case-by-case basis [16,17,19].

The hormonal profile and molecular abnormalities specific to the ectopic endo-metrium are the key elements that characterize the pathogenesis of endometriosis. The present study aimed to evaluate the molecular profile of ectopic endometrium tissues by analyzing the expression of ER, PR, anti-apoptotic Bcl-2 protein and Ki-67 antigen expression based on rASRM stage and symptoms.

## 2. Results

According to the rASRM classification, the 14 patients were classified as follows: four patients (28.6%) with rASRM stage II, six patients (42.9%) with rASRM stage III, and four patients (28.6%) with rASRM stage IV. The distribution of patients according to the rASRM classification did not show a significant trend (*p* = 0.751).

**Figure 1:**
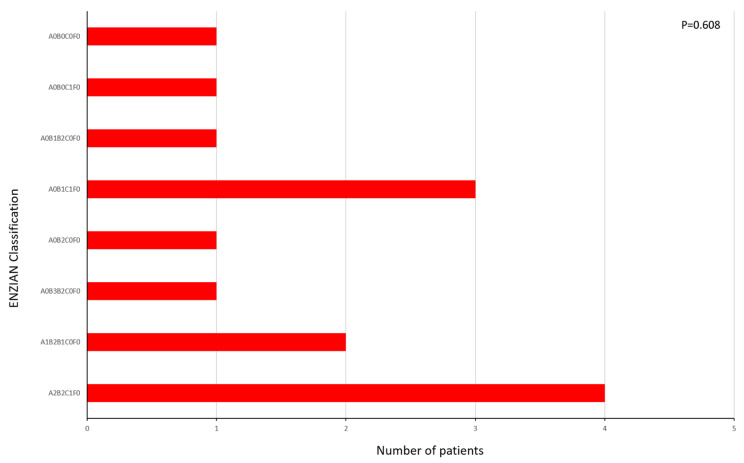
Distribution of patients according to the ENZIAN classification.

The distribution of patients according to the ENZIAN classification is shown in Figure 1, and there were no statistically significant differences in the distribution of patients (*p* = 0.608).

**Figure 2:**
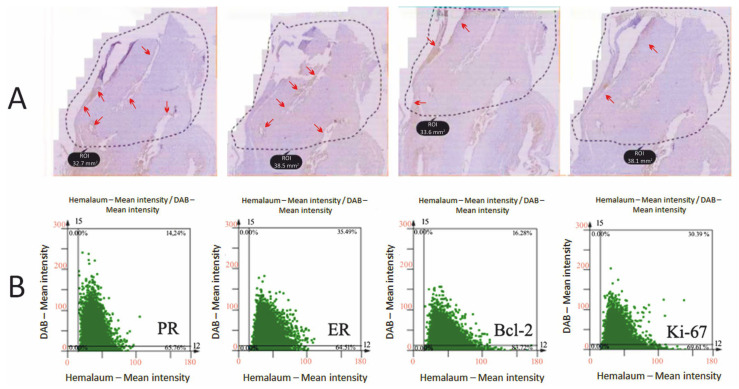
The quantitative analysis of the immunohistochemical expression of PR, ER, Bcl-2 and Ki-67 markers. (**A**)—Defining regions of interest. (**B**)—Dot-plots generated on the region of interest defining the DAB-positive populations. Arrows identify the areas with positive signals.

The quantitative analysis of the immunohistochemical expression of PR, ER, Bcl-2, and Ki-67 markers in a patient with endometriosis stage III rASRM is shown in Figure 2.

Table 1 shows the distribution of the epithelial and stromal ER biomarkers, based on the rASRM stage. Data are presented as the mean value and range. The calculated *p*-values show the significance of the distribution of mean values based on rASRM stage. The average values of epithelial and stromal ER did not show a significant difference in distribution based on rASRM stage.

The distribution of the epithelial and stromal PR biomarkers based on rASRM stage is shown in Table 2. Data are presented as the mean value and range. The calculated *p*-values show the significance of the distribution of mean values based on rASRM stage. The mean values of epithelial and stromal PR did not show a significant difference in distribution based on rASRM stage.

Table 3 summarizes the expression of the epithelial and stromal Bcl-2 based on rASRM stage. Data are presented as the mean value and range. The calculated *p*-values show the significance of the distribution of mean values based on rASRM stage. The mean values of epithelial and stromal Bcl-2 did not show a significant difference in distribution based on rASRM stage.

Table 4 summarizes the expression of the epithelial and stromal Ki-67 based on rASRM stage. Data are presented as the mean value and range. The calculated *p*-values show the significance of the distribution of mean values based on rASRM stage. The mean values of epithelial and stromal Ki-67 did not show a significant difference in distribution based on rASRM stage.

Table 5 shows the ratio of positive/negative cases for epithelial ER, stromal ER, epithelial PR, stromal PR, epithelial Bcl-2, stromal Bcl-2, epithelial Ki-67, and stromal Ki-67, based on rASRM stage. The calculated *p*-values show the significance of the distribution of the ratio of positive/negative cases for all the biomarkers analyzed. There were no significant differences in the distribution of the ratio of any of the markers based on stage.

Table 6 shows the correlations among the analyzed biomarkers from the ectopic endometrium of patients in the studied group.

Epithelial ER was significantly positively correlated with stromal ER (*p* = 0.007), epithelial PR (*p* < 0.001), and stromal PR (*p* = 0.027). Epithelial ER was not correlated with epithelial Bcl-2 (*p* = 0.277) or stromal Bcl-2 (*p* = 0.243).

Stromal ER was positively correlated with epithelial PR (*p* = 0.040) and stromal Ki-67 (*p* = 0.011). Stromal ER showed a trend toward a positive correlation (although not significantly) with epithelial Bcl-2 (*p* = 0.067) and stromal Bcl-2 (*p* = 0.055).

Finally, epithelial Bcl-2 was positively correlated with stromal Bcl-2 (*p* < 0.001). Epithelial Ki-67 was positively correlated with stromal Ki-67 (*p* = 0.003) (Table 6).

Table 7 shows the correlations among the personal history of endometriosis, history of surgery for endometriosis, and symptoms (dysmenorrhea, dyspareunia, chronic pelvic pain, or gastrointestinal symptoms) with Bcl-2 and Ki-67 expression in the ectopic endometrium.

Epithelial Bcl-2, stromal Bcl-2, epithelial Ki-67 and stromal Ki-67 were not statistically significantly correlated with a personal history of endometriosis or with a history of surgery for endometriosis. Similarly, there were no correlations to the epithelial or stromal expression of these markers with the presence of symptoms (Table 7).

**Figure 3:**
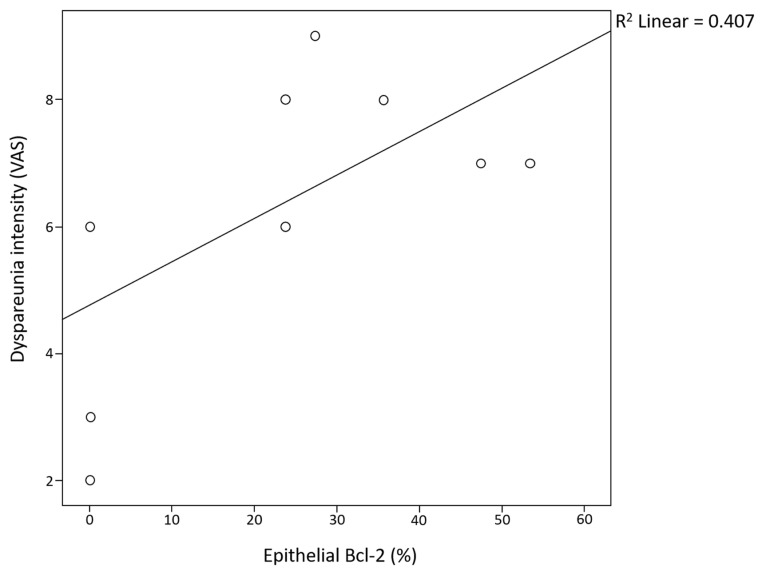
Correlation between epithelial Bcl-2 biomarker values and dyspareunia intensity.

Epithelial Bcl-2 was positively correlated with the intensity of dyspareunia in the 10 patients with dyspareunia (Figure 3).

Among the 14 patients included in the present study, five patients (35.71%) had no history of surgery for endometriosis. The other nine patients (64.29%) had at least one surgery for endometriosis, so they had recurrent endometriosis. Of these, five patients (35.71%) had one previous surgery, three patients (21.4%) had two previous surgeries, and one patient (7.1%) had six previous surgeries for endometriosis.

The mean value of epithelial Ki-67 in patients with recurrent endometriosis (6.08 ± 10.58%) was lower compared with the mean value of epithelial Ki-67 in patients without recurrent endometriosis (7.87 ± 10.27%), although the difference was not significant (*p* = 0.765). The mean value of stromal Ki-67 in patients with recurrent endometriosis (13.56 ± 20.18%) was higher than the mean value of stromal Ki-67 in patients without recurrent endometriosis (9.27 ± 16.02%) (*p* = 0.691).

## 3. Discussion

The evaluation of a patient with endometriosis includes a complete history, an assessment of symptoms (which can often be non-specific), and at least one imaging method [5]. Transvaginal ultrasound is considered the first-line imaging technique in endometriosis [20]. Magnetic resonance imaging (MRI) is considered the second-line imaging modality and is recommended if transvaginal ultrasound is inconclusive or if additional endometriotic lesions are suspected [20].

Infertility is one of the main symptoms present in patients with endometriosis and occurs through the synergy of several mechanisms: reduction of normal ovarian tissue, alteration of the number or quality of oocytes, inflammatory characteristics of the peritoneal fluid, and reduced endometrial receptivity [21]. Given that the vast majority of patients with endometriosis want to achieve a pregnancy, assisted human reproduction plays a central role in the personalized management of these patients, both before surgery through oocytes/embryos cryopreservation techniques or through in vitro fertilization after surgical treatment [22,23]. The influence of endometriosis on pregnancies obtained through in vitro fertilization has been analyzed by several studies, which concluded that the morphology of oocytes and embryos is not affected by this disease [21,23]. Patients with endometriosis can confidently use assisted human reproduction techniques, because the neonatal prognosis and neuro-psycho-motor development of children obtained through these methods have not been associated with the occurrence of neurological diseases (autism spectrum disorders), impaired cognitive performance or the occurrence of infantile cerebral palsy [24].

Even if serum estrogen levels in a patient with endometriosis are similar to a healthy woman, the changes induced by estrogen play a central role in the development of endometriosis [2]. The local synthesis of estrogen associated with increased activity of ERs in endometriotic lesions results in the appearance of a hyperestrogenic state in patients with endometriosis [2]. Excessively high local levels of estradiol result in a decrease in apoptosis in the endometriotic tissue (both at the stromal and epithelial level) when compared with the eutopic endometrium [2]. Estrogen is an important mediator of chronic inflammation, determining endometriotic cell survival and tissue growth [2,14]. Estradiol (a biologically active form of estrogen) plays a critical role in the reconstructive (proliferative) phase of the endometrium following menstruation, through ER1 (also known as ER-α) and ER2 (also known as ER-β) [10,14]. ER1 and ER2 are genetically encoded by two distinct genes [10,14]. Aromatase stimulates the conversion of ovarian androstenedione to estrone, and 17β-hydroxysteroid dehydrogenase (17β-HSD)1 stimulates the conversion of estrone to estradiol [11]. 17β-HSD2 intervenes in the luteal phase, being stimulated by progesterone, and stimulates the conversion of estradiol to estrone (a less potent form of estrogen) [2,11].

Progesterone is primarily secreted during the secretory phase of the menstrual cycle and acts on the endometrium through PR-A and PR-B, exerting a dual role: inhibiting the action of estrogen (antiestrogenic effect) and preparing the endometrium for implantation [10,11]. Both forms of progesterone receptors are genetically encoded by the same gene, although the PR-A splice variant is 164 amino acids shorter than PR-B [10,14]. PR expression is stimulated by estrogen through ER 1, and subsequently, progesterone receptors inhibit the expression of ER 1, thus creating a feedback mechanism that maintains hormonal homeostasis [14]. The serum levels of progesterone in patients with endometriosis are similar to those of healthy women [2]. The ectopic endometrium in endometriotic lesions is characterized by an increase in ER 2 concentration (~140× higher than the eutopic endometrium of healthy women) and a reduction in ER 1 levels; ER 2 in endometriotic stromal cells saturates the implantation loci of ER 1 precursors and suppresses ER 1 levels, with a subsequent increase in the ER 2/ER 1 ratio [10,14]. Given that estrogen increases the expression of progesterone receptors through ER 1, the low levels of ER 1 in endometriotic lesions may explain the reduced level of progesterone receptors in the ectopic endometrium [14].

Endometriosis implants are characterized by increased levels of aromatase and 17β-HSD1, associated with lower levels of 17β-HSD2 (in response to the reduced levels of progesterone receptors), and therefore, an increased level of estradiol is observed in patients with endometriosis, both in the eutopic and ectopic endometrium [2,11].

Endometriosis is associated with progesterone resistance, characterized by the inability of the endometrial tissue to respond adequately to the action of progesterone; this state manifests due to a failure in activation of progesterone receptors and an inability to use the available progesterone [11,14]. Ectopic endometrium from endometriotic implants exhibited reduced PR expression levels, with an absence of PR-B and a very low level of PR-A, compared to normal endometrium [25]. Additionally, alterations of the mediators and of the PR regulatory molecules contributed to progesterone resistance in endometriosis [14]. Progesterone resistance was associated both with the proliferation of endometriotic lesions and with reduced endometrial receptivity [2,14].

The hormonal treatment of endometriosis consists primarily of the following drugs: anti-estrogens, progestins, estrogen, and PR modulators [15]. Hormonal treatment with progestins and combined oral contraceptives is ineffective in almost one-third of patients with endometriosis for incompletely understood reasons [15]. Considering that the mechanism of action of these therapies is based on estrogen and progesterone receptors, there is a possibility that the expression of the receptors of these hormones is altered in endometriosis, which leads to the absence of a therapeutic response [15].

The biochemical analysis of the ectopic endometrium in the present study highlighted the presence of the studied biomarkers (ER, PR, Bcl-2, and Ki-67), both at the epithelial and stromal levels, in the samples from endometriotic lesions. The presence of ER at the epithelial level (14.7% in rASRM stage II, 16.8% in rASRM stage III, and 25.2% in rASRM stage IV) and at the stromal level (28.2% in rASRM stage II, 7.5% in rASRM stage III, and 45.2% in rASRM stage IV) in the analyzed group demonstrated the sensitivity and reactivity of the ectopic endometrium to the estrogenic stimulus, corroborating previous studies [26,27].

The studied group exhibited epithelial PR expression in 13.2% in rASRM stage II, 44.1% in rASRM stage III, and 52.9% in rASRM stage IV; stromal PR expression was found in 59.8% in rASRM stage II, 54.4% in rASRM stage III and 70.7% in rASRM stage IV. In general, the studied group was characterized by a lower expression of ER compared to PR; these results contradict previous studies, where reduced expression of PR and moderately increased ER expression was observed [25,26]. In the present group, PR expression was increased, particularly in rASRM stages III and IV; the results obtained contradict a previous study, where reduced PR expression was observed in the ectopic endometrium [25]. The results of the present study may indicate a superior therapeutic response to progesterone treatment in cases with increased expression of PR; cases with resistance to progesterone treatment may be explained by the presence of reduced PR expression in endometriotic lesions.

The different values and the random distribution of ER and PR related to the disease stages may indicate that there are multiple types of hormonally mediated endometriosis lesions, with different hormonal sensitivities, or that changes in hormonal regulation occur as the lesions progress under the influence of different environmental factors [28].

The statistical analysis of the molecular profile in the studied group showed that the levels of epithelial ER were directly proportional to epithelial PR and stromal PR expression, and the expression of stromal ER was correlated with epithelial PR expression. These results contradict the findings of a previous study, where it was shown that increased expression of ER in the ectopic endometrium was associated with decreased expression of PR [25].

Apoptosis or programmed cell death is a process that has a crucial role in human physiology, tissue homeostasis, and the clearance of compromised or infected cells [16]. Apoptosis is controlled by regulatory proteins that maintain homeostasis by regulating pro-apoptotic and anti-apoptotic effects at the tissue level [16]. Bcl-2 is an important member of the Bcl-2 protein family; its gene is located on chromosome 18, and the protein functions to promote cell survival through its anti-apoptotic role [16,29]. Endometriosis is characterized by aberrant cell proliferation of the ectopic endometrial tissue associated with dysregulation of the apoptosis mechanisms [16]. Combined oral contraceptives administered to patients with endometriosis leads to a significant decrease in cell proliferation and stimulation of apoptosis in the eutopic endometrium, and a reduction in Bcl-2 levels is observed [16].

In the present study, positive epithelial Bcl-2 expression was found in 13.6% in rASRM stage II, 17.8% in rASRM stage III, and 23.7% in rASRM stage IV; positive stromal Bcl-2 expression was observed in 15.3% in rASRM stage II, 20.0% in rASRM stage III, and 26.7% in rASRM stage IV. These results agree with a study published by Nasu et al. [16]. The expression of the Bcl-2 protein both at the epithelial and at the stromal level is directly proportional to the stage of the disease, but there was no statistically significant correlation. This distribution may indicate an increased level of cell proliferation as the stage of endometriosis advances, and implicitly, the potential value of Bcl-2 as a biomarker of aggressiveness. Additional studies with a larger cohort of patients are required to investigate this hypothesis.

There was no significant correlation between epithelial ER with epithelial or stromal Bcl-2 expression. Stromal ER was associated (although the association was not significant) with epithelial and stromal Bcl-2 expression. These results contradict a previous study, which revealed a direct correlation between the increased levels of ER and the expression of Bcl-2 [16]. Expanding this study using a larger group of patients is required to determine whether there is a significant correlation between stromal ER and epithelial Bcl-2 or stromal Bcl-2.

The Ki-67 antigen is a nuclear protein specifically associated with cell proliferation [18]. The Ki-67 antigen consists of two subunits, a 345 and 395 kDa subunit, and it is coded by a gene located on chromosome 10 [17]. The Ki-67 antigen is present in all active phases of the cell cycle, both in interphase (G1, S, and G2) and during division, and is associated with mitosis [18]. This pattern of Ki-67 antigen expression during cell cycle phases (both in normal and tumor cells) is the basis for using Ki-67 as an indicator of proliferation for a specific cell population and is frequently used to calculate the proliferation index or staging of tumors [17,18]. The Ki-67 proliferation index is used to estimate tumor aggressiveness, prognosis, survival, and recurrence rate [17,19]. The proliferation of endometriosis is directly correlated with progression, which involves the development of complex tumor lesions and the ability of endometriotic lesions to invade adjacent tissues [18]. Consequently, multiple studies have evaluated the relationship between the Ki-67 antigen and endometriotic lesions [17].

In the present study, positive expression of epithelial Ki-67 was found in 3.8% in rASRM stage II, 8.1% in rASRM stage III, and 7.6% in rASRM stage IV; positive stromal Ki-67 expression was observed in 9.3% in rASRM stage II, 8.4% in rASRM stage III, and 20.1% in rASRM stage IV. Although Ki-67 expression was identified in all stages of endometriosis, there was not a significant correlation between Ki-67 expression and rASRM stage; these results contradict previous studies, which showed a directly proportional relationship between Ki-67 expression and endometriotic stage [18,19,30]. A possible explanation for the lack of correlation between Ki-67 and the endometriotic stage in the analyzed group may be that Ki-67 is an indicator of dissemination potential and prognosis, as shown previously [17,19]. The increased (but insignificant) expression of Ki-67 concerning disease stage highlights the need for future studies to analyze its value as a biomarker of aggressiveness and prognosis of endometriosis.

Analysis revealed the absence of a statistically significant correlation between the expression of Bcl-2 (epithelial or stromal) and Ki-67 (epithelial or stromal) biomarkers and a personal history of endometriosis, previous surgeries for endometriosis or the presence of symptoms (dysmenorrhea, dyspareunia, pelvic pain chronic, or gastrointestinal symptoms). Specific analysis of the relationship between Ki-67 and recurrent endometriosis did not reveal a statistically significant correlation. These results contradict a previous study, in which Ki-67 was an indicator of the risk of endometriosis recurrence [17].

Even though the expression of ER, PR, Bcl-2, and Ki-67 was identified in the ectopic endometrium in all stages of endometriosis, there were no significant correlations between the levels of these biomarkers and the rASRM stage. A possible explanation for these results may be the fact that the endometriotic lesions present in the patients were in different stages of evolution, as well as the fact that not all endometriotic lesions have the same capacity for progression.

Although this is an old study, conducted between January and December 2015, there is still significant interest in understanding the clinical and molecular characteristics, as well as the management, of endometriosis [31]. The biomarkers analyzed in the present study were also evaluated in other studies [31]. The study published by Matasariu et al. analyzed the expression of the biomarkers evaluated by us (ER, PR, Ki-67 and Bcl-2) and also osteopontin and vascular endothelial growth factor in patients with ovarian endometrioma with or without progesterone treatment; that study concluded that progesterone treatment increased the serum level of osteopontin, the tissue expression of PR and Bcl-2, and reduced the serum level of vascular endothelial growth factor and the tissue expression of Ki-67 [31].

In addition to the biochemical profile of the ectopic endometrium of patients with endometriosis, nutrition also has a significant role in long-term management [32]. Including a dietitian in the multidisciplinary therapeutic team of these patients can lead to a significant reduction of the painful symptoms [32]. Pain in endometriosis is improved due to the anti-inflammatory effects of omega-3 fatty acids and anti-oxidant vitamins (C, D and E) [32].

A major limitation of the present study is the relatively low number of patients included, which could influence the generalizability of the results. The low number of patients enrolled in this study was directly determined by the high cost of the biochemical tests performed. Another limitation of the study is represented by the biomarker evaluation method, which has not analyzed separately (due to technical and financial limitations) the estrogen and progesterone receptor subtypes (ER-α and ER-β, and PR-A and PR-B, respectively), considering that these forms exert different roles in the development of the disease [10,11,14].

Further research on the biochemical profile of the ectopic endometrium on a larger number of patients is necessary to clarify the relationship between these biomarkers and the clinical management of patients with endometriosis. In addition, subsequent studies should include a separate analysis of estrogen receptor subtypes (ER-α and ER-β) and progesterone receptor subtypes (PR-A and PR-B). Also, considering that endometriosis is a chronic inflammatory pathology, the evaluation of chronic inflammation associated with endometriosis represents a goal of future research [1,2].

## 4. Materials and Methods

### 4.1. Patient Data and Selection Criteria

The present study was a prospective study conducted over a period of 1 year (January–December 2015). The study included 14 patients with endometriosis admitted to the “Sf. Pantelimon” Clinical Emergency Hospital (Bucharest, Romania). The following data were collected for each patient: personal history of endometriosis, previous surgeries for endometriosis, and the presence/absence of symptoms (dysmenorrhea, dyspareunia, chronic pelvic pain, or gastrointestinal symptoms). The intensity of dysmenorrhea and dyspareunia was quantified using a visual analog scale (VAS). A patient was considered to have gastrointestinal symptomatology if she confirmed the presence of ≥1 symptoms (painful defecation, presence of blood in the feces, bloating, constipation, diarrhea, or change in the appearance of the feces); other gastrointestinal pathologies were excluded by differential diagnosis.

The inclusion criteria were as follows: clinical symptomatology and ultrasound investigations highly suggestive of endometriosis, providing informed consent, endometriosis treatment by laparoscopic surgery, and intraoperative collection followed by biochemical analysis of ectopic endometrial tissue samples. The exclusion criteria were as follows: treatment with hormonal preparations for endometriosis in the last 6 months, autoimmune diseases, genetic diseases, infectious pathology in the pelvic–genital sphere, diabetes mellitus, or malignancies. All patients included in the study have given their verbal consent for participation.

### 4.2. Surgical Procedure and Tissue Collection

All enrolled patients underwent laparoscopic surgical treatment to excise the endometriotic lesions. The final diagnosis of endometriosis was confirmed by pathological examination of the lesions. Following the surgical procedure, the rASRM and ENZIAN classification of each case was performed [7,8]. Ectopic endometrial samples from endometriotic lesions collected from each patient were further analyzed by immunohistochemistry (IHC) to determine the expression of the biomarkers of interest (hormonal biomarkers, ER and PR; apoptotic and proliferative biomarkers, Bcl-2 and Ki-76) in the stroma and glandular epithelium. The expression of biomarkers in the ectopic endometrium was comparatively analyzed according to the rASRM stage. The pathological examination defined the regions of interest of the biopsy samples such that the IHC analysis for the biomarkers was performed on tissue fragments of ectopic endometrium.

### 4.3. IHC

The IHC analysis of the ectopic endometrium was performed by the Anatomic-Pathology Department of “Colentina” Clinical Hospital (Bucharest, Romania).

Paraffin embedding was preceded by fixation, which stabilizes and preserves the structure and cellular components to maintain them as close as possible to their original, living state [33]. The formalin-fixed paraffin-embedded tissue blocks were cut into 5 μm sections. Staining was performed either manually or automatically using a Leica Bond III device (Leica Microsystems GmbH, Wetzlar, Germany). All procedures were performed in accordance with the Medical Practical Guides for Anatomic-Pathology (Romanian Ministry of Health Order no. 1217/16th of September 2010). Briefly, the samples were deparaffinized using a standard protocol. Endogenous peroxidase activity was quenched using 3% H_2_O_2_ for 15 min followed by epitope retrieval by incubating the samples in citrate buffer at 98 °C for 20 min. Tissues were blocked in a solution of phosphate-buffered saline (PBS) containing 1% bovine serum albumin (BSA), 0.1% gelatin, 0.05% Tween 20, and 0.1% Triton X-100 for 30 min at room temperature. The primary antibodies used were: anti-ER (rabbit monoclonal, Cell Marque www.cellmarque.com accessed on 5 January 2015, clone SP1, 1:60, 4 °C overnight), anti-PR (mouse monoclonal, Leica Novocastra https://www.leicabiosystems.com/ihc-ish/novocastra-antibodies accessed on 5 January 2015, clone 16, 1:100, 1 h at room temperature), anti-Bcl-2 (mouse monoclonal, Leica Novocastra https://www.leicabiosystems.com/ihc-ish/novocastra-antibodies accessed on 5 January 2015, clone Bcl-2/100D5, 1:160, 1 h at room temperature), and anti-Ki-67 (mouse monoclonal, Leica Novocastra https://www.leicabiosystems.com/ihc-ish/novocastra-antibodies accessed on 5 January 2015, clone MM1, 1:100, 1 h at room temperature). Antibody incubations were performed in a humid chamber at room temperature. Sections were then incubated with the corresponding secondary antibodies labeled with streptavidin and further developed using diaminobenzidine (DAB) (Novolink kit, Leica Microsystems GmbH) following a 5–15 min incubation. Before the incubation with both the primary and secondary antibodies, tissue samples were washed with Tris-buffered saline (TBS) solution, pH 7.4, two times, 10 min each. Finally, the samples were washed with water and stained further to contrast with Meyer’s hematoxylin. After drying, the samples were mounted and examined using a Zeiss Axio Imager 2 microscope (Carl Zeiss GmbH, Oberkochen, Germany).

### 4.4. Biomarker Evaluation

Immunohistochemical analysis of ER, PR, and Ki-67 in the stroma and glandular epithelium was quantified using a semi-quantitative scoring method similar to that described by Allred et al. [34]. The score was based on the percentage of positive cells and the intensity of the staining reaction. The percentage of positive cells was scored from 0–5 as follows: 0, no staining; 1, <1% positive cells; 2, 1–10% positive cells; 3, 11–33% cells positive; 4, 34–66% positive cells; and 5, 67–100% positive cells. Staining intensity was scored from 0–3 as follows: 0, no staining; 1, low intensity staining; 2, moderate intensity staining; and 3, high intensity staining. The final score was obtained by adding the two scores. Each case was considered negative or positive for the respective biomarker based on the value of the final score; a final score between 0–2 was considered negative, and a final score between 3–8 was considered positive; cases with no immunohistochemical reaction were considered negative.

Immunohistochemical expression of Bcl-2 protein in the stroma and glandular epithelium was assessed using a semi-quantitative score similar to that described by Suzuki et al. [35]. The score was based on the percentage of positive cells. The proportion of cells stained was scored as follows: 0, <1% positive cells; 1, 1–25% positive cells; 2, 26–50% positive cells; 3, 51–75% positive cells; and 4, >75% positive cells. Cases with a score of 0 were considered negative, and those with a score between 1–4 were considered positive.

The cytometric analysis of the images was performed using a TissueFAXSiPlus technological platform (TissueGnostics, Vienna, Austria), which allowed for the scanning and reconstruction of preparations mounted on microscopy slides and marked for IHC using a PixelLink camera (color, for visible light). All images were acquired using the same settings (exposure time, objective, sensitivity) using a Zeiss Axio Observer Z1 Microscope (Carl Zeiss GmbH, Oberkochen, Germany). The quantitative analysis of the expression of the proteins of interest was performed using the HistoQuest (IHC) software (version 3.5.3.017) module based on the detection of stained cell nuclei.

### 4.5. Statistical Analysis

Statistical analysis was performed using IBM SPSS version 23.0 (IBM Corp.). *p* < 0.05 was considered to indicate a statistically significant difference. Data are presented as the means ± SDs for quantitative variables and as frequencies (%) for qualitative variables. To compare the epithelial/stromal ER, PR, Bcl-2, and Ki-67 expression based on rASRM stage, the analysis of variance (ANOVA) test was used. The distributions of the ratio of positive/negative cases for epithelial ER, stromal ER, epithelial PR, stromal PR, epithelial Bcl-2, stromal Bcl-2, epithelial Ki-67, and stromal Ki-67, depending on rASRM stage, was analyzed using a chi-square test (χ2) test. To study the correlations between analyzed biomarkers, a Spearman’s rank correlation test (−1 ≤ rs ≤ +1) was used.

## 5. Conclusions

The ectopic endometrium from endometriotic lesions showed variable expression of ER, PR, Bcl-2, and Ki-67. The expression of these biomarkers did not correlate with the stage of endometriosis. In the analyzed group, the expression level of progesterone receptors was higher than the expression level of estrogen receptors.

The expression of Bcl-2 and Ki-67 was not correlated with a personal history of endometriosis, with previous surgical interventions for endometriosis or painful symptoms (dysmenorrhea, dyspareunia, chronic pelvic pain, or gastrointestinal symptoms). Ki-67 was not correlated with recurrent endometriosis. Epithelial ER expression was not correlated with epithelial or stromal Bcl-2 levels. Stromal ER showed a trend toward a correlation with epithelial and stromal Bcl-2 expression (though not significant). Epithelial Bcl-2 levels were significantly correlated with the intensity of dyspareunia.

The variable expression of ER, PR, Bcl-2, and Ki-67 provide a basis for further studies on individualized hormonal medical treatment and for identifying aggressive forms of endometriosis with a high risk of recurrence. The complete characterization of the biochemical profile of endometriosis lesions in this study contributes to the identification of optimal and personalized long-term treatment regimens for patients with endometriosis.

## Figures and Tables

**Table 1:** Distribution of patients according to the mean value of epithelial/stromal ER and rASRM stage.

	Biomarker	Epithelial ER		Stromal ER	
rASRM Stage					
rASRM stage II		Mean ± SD	range	Mean ± SD	range
		14.7 ± 12.0%	0–90%	28.2 ± 23.0%	0–100%
rASRM stage III		Mean ± SD	range	Mean ± SD	range
		16.8 ± 21.2%	0–90%	7.5 ± 9.5%	0–100%
rASRM stage IV		Mean ± SD	range	Mean ± SD	range
		25.2 ± 20.5%	0–90%	45.2% ± 36.9%	0–100%
*p*-value		*p* = 0.710		*p* = 0.084	

**Table 2:** Distribution of patients according to the mean value of epithelial/stromal PR and rASRM stage.

	Biomarker	Epithelial PR		Stromal PR	
rASRM Stage					
rASRM stage II		Mean ± SD	range	Mean ± SD	range
		13.2 ± 10.8%	0–100%	59.8 ± 40.1%	5–100%
rASRM stage III		Mean ± SD	range	Mean ± SD	range
		44.1 ± 42.6%	0–100%	54.4% ± 43.1%	5–100%
rASRM stage IV		Mean ± SD	range	Mean ± SD	range
		52.9 ± 38.5%	0–100%	70.7 ± 41.5%	5–100%
*p*-value		*p* = 0.285		*p* = 0.836	

**Table 3:** Distribution of patients according to the mean value of epithelial/stromal Bcl-2 and rASRM stage.

	Biomarker	Epithelial Bcl-2		Stromal Bcl-2	
rASRM Stage					
rASRM stage II		Mean ± SD	range	Mean ± SD	range
		13.6 ± 11.1%	0–90%	15.3 ± 12.5%	0–90%
rASRM stage III		Mean ± SD	range	Mean ± SD	range
		17.8 ± 22.5%	0–90%	20.0 ± 25.3%	0–90%
rASRM stage IV		Mean ± SD	range	Mean ± SD	range
		23.7 ± 19.4%	0–90%	26.7 ± 25.0%	0–90%
*p*-value		*p* = 0.760		*p* = 0.776	

**Table 4:** Distribution of patients according to the mean value of epithelial/stromal Ki-67 and rASRM stage.

	Biomarker	Epithelial Ki-67		Stromal Ki-67	
rASRM Stage					
rASRM stage II		Mean ± SD	range	Mean ± SD	range
		3.8 ± 5.3%	0–35%	9.3 ± 13.2%	0–90%
rASRM stage III		Mean ± SD	range	Mean ± SD	range
		8.1 ± 10.2%	0–35%	8.4 ± 14.4%	0–90%
rASRM stage IV		Mean ± SD	range	Mean ± SD	range
		7.6 ± 14.9%	0–35%	20.1 ± 28.4%	0–90%
*p*-value		*p* = 0.811		*p* = 0.616	

**Table 5:** Distribution of patients according to epithelial ER, stromal ER, epithelial PR, stromal PR, epithelial Bcl-2, stromal Bcl-2, epithelial Ki-67, stromal Ki-67 positive/negative cases and rASRM stage.

	Biomarker (pos./ neg.)	Epithelial ER (pos./ neg.)	Stromal ER (pos./ neg.)	Epithelial PR (pos./ neg.)	Stromal PR (pos./ neg.)	Epithelial Bcl-2 (pos./ neg.)	Stromal Bcl-2 (pos./ neg.)	Epithelial Ki-67 (pos./ neg.)	Stromal Ki-67 (pos./ neg.)
rASRM Stage									
rASRM stage II		1/3	3/1	3/1	4/0	2/2	2/2	3/1	3/1
rASRM stage III		2/4	4/2	4/2	6/0	3/3	3/3	4/2	4/2
rASRM stage IV		1/3	3/1	3/1	4/0	2/2	2/2	3/1	3/1
*p*-value		*p* = 0.943	*p* = 0.943	*p* = 0.943		*p* = 1.000	*p* = 1.000	*p* = 0.943	*p* = 0.943

pos. = positive; neg. = negative.

**Table 6:** Correlations among the analyzed biomarkers from the ectopic endometrium.

	1	2	3	4	5	6	7	8	9
1. rASRM stage									
2. Epithelial ER	0.431								
3. Stromal ER	0.400	0.007 *							
4. Epithelial PR	0.130	0.000 *	0.040 *						
5. Stromal PR	0.814	0.027 *	0.405	0.060					
6. Epithelial Bcl-2	0.451	0.277	0.067	0.297	0.260				
7. Stromal Bcl-2	0.470	0.243	0.055	0.265	0.220	0.000 *			
8. Epithelial Ki-67	0.611	0.366	0.248	0.095	0.530	0.353	0.248		
9. Stromal Ki-67	0.427	0.213	0.011 *	0.151	0.920	0.221	0.139	0.003 *	-

* *p* < 0.05.

**Table 7:** Correlations among a personal history of endometriosis, previous surgeries for endometriosis, symptomatology and molecular biomarkers from the ectopic endometrium.

	1	2	3	4	5	6	7	8	9	10
1. Personal history of endometriosis										
2. Previous surgeries for endometriosis	0.025 *									
3. Dysmenorrhea	0.190	0.447								
4. Dyspareunia	0.519	0.302	0.549							
5. Chronic pelvic pain	0.746	0.361	0.288	0.249						
6. Gastrointestinal symptoms	0.630	0.124	0.549	0.156	0.803					
7. Epithelial Bcl-2	0.224	0.286	0.311	0.378	0.728	0.056				
8. Stromal Bcl-2	0.177	0.256	0.332	0.397	0.689	0.068	0.000 *			
9. Epithelial Ki-67	0.765	0.651	0.068	0.837	0.927	0.264	0.353	0.248		
10. Stromal Ki-67	0.691	0.467	0.164	0.349	0.853	0.255	0.221	0.139	0.003 *	-

* *p* < 0.05.

## Data Availability

The data from this study can be provided on request by the corresponding author.

## Abbreviations

The following abbreviations are used in this manuscript:

17β-HSD	17β-hydroxysteroid dehydrogenase
ANOVA	analysis of variance
Bcl-2	B-cell lymphoma-2
BSA	bovine serum albumin
DAB	diaminobenzidine
ER	estrogen receptor
IHC	immunohistochemistry
MRI	magnetic resonance imaging
PBS	phosphate buffered saline
PR	progesterone receptor
rASRM	revised American Society for Reproductive Medicine
TBS	Tris-buffered saline
VAS	visual analog scale
